# Evolutionary Digital Twin for Oil and Gas Pipelines: A Cognitive Multi-Agent Framework with Continuous Feedback Learning

**DOI:** 10.3390/s26103219

**Published:** 2026-05-19

**Authors:** Ning Shi, Zixuan Li, Qiujuan Li, Jing Zhang, Liangliang Li, Qiaofei Sun, Sijia Liu, Zheng Wang

**Affiliations:** 1 Intelligent Research Center, PipeChina Institute of Science and Technology, Tianjin 300450, China; shining02@pipechina.com.cn (N.S.); zhangjing33@pipechina.com.cn (J.Z.); lill08@pipechina.com.cn (L.L.); sunqf01@pipechina.com.cn (Q.S.); liusj23@pipechina.com.cn (S.L.); 2 School of Artificial Intelligence, Tianjin University, Tianjin 300072, China; lizix@tju.edu.cn

**Keywords:** multi-agent system (MAS), digital twin, large language models (LLM), physics-informed neural networks (PINN), multi-sensor data fusion, dynamic risk assessment, pipeline integrity management, continuous learning

## Abstract

The structural integrity and risk management of long-distance oil and gas pipelines are critically challenged by multi-source data heterogeneity, complex multi-physics degradation mechanisms, and the dynamic nature of operational environments. Traditional monolithic artificial intelligence models struggle with cross-domain knowledge fusion and often suffer from historical context forgetting over decades-long infrastructure lifecycles. To address these bottlenecks, this paper proposes an evolutionary digital twin framework driven by a collaborative architecture between small specialized models and a large general model. Specifically, the framework encapsulates physics-informed models (e.g., corrosion prediction and geohazard evaluation) as domain expert agents to guarantee rigorous numerical computation at the edge, keeping sensitive operational data strictly localized. To synthesize conflicting localized risks, a locally deployed, privacy-preserving large language model acts as a central cognitive hub. This hub utilizes external knowledge retrieval and structured reasoning to formulate transparent, multi-objective intervention strategies. Furthermore, a continuous feedback learning mechanism is introduced to capture tacit expert knowledge. By formalizing human operational interventions into historical memory and employing parameter stabilization techniques, the system dynamically updates its knowledge base while effectively mitigating catastrophic forgetting. Ultimately, the proposed framework provides a reliable and privacy-compliant methodology, significantly enhancing the interpretability and predictive foresight of pipeline integrity management.

## 1. Introduction

Long-distance oil and gas pipelines are the core of the modern energy supply chain [1]. However, continuous network expansion and asset aging present severe management challenges. The industry must accelerate its shift toward resilient and sustainable operations [2,3]. This transition to sustainable infrastructure closely aligns with the emerging Digital Twin as a Service (DTaaS) paradigm [4]. As pipelines age, the cumulative effects of environmental stress and operational wear become highly pronounced.

**Figure 1:**
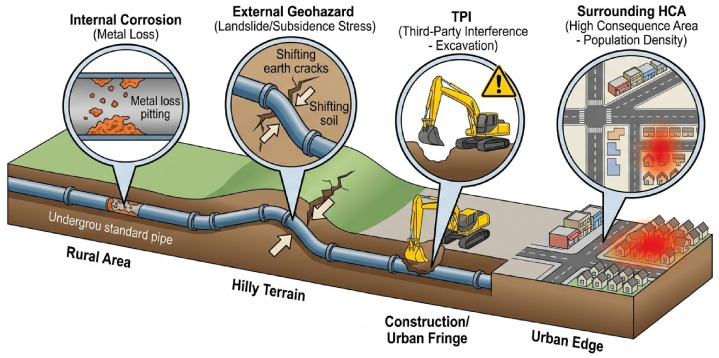
Multi-Dimensional Risk Scenarios in Long-Distance Pipelines. This schematic illustrating a 3D cutaway view of the pipeline stretches across varied terrain, highlighting core risk categories: internal corrosion with metal loss pitting, external geohazard landslide stress, surface third-party interference from digging machinery, and surrounding high population density areas.

Complex internal degradation and external environmental stressors continuously threaten pipeline structural integrity. A typical network traverses highly heterogeneous terrains. As illustrated in Figure 1, this exposes pipelines to diverse dynamic risks. These risks include electrochemical corrosion, geological hazards, and third-party mechanical interference [5].

A severe data silo problem compounds these physical threats [6]. Modern pipelines utilize advanced multi-modal sensor networks. However, the generated data remains fragmented across different departments and spatial reference systems [7]. This fragmentation prevents the formation of a holistic risk profile. Table 1 summarizes recent high-profile disasters. These incidents illustrate the systemic failure of traditional risk management paradigms.

Current risk assessment relies heavily on subjective engineering experience. This reliance fosters a reactive maintenance culture. The disaster cases highlight critical technical bottlenecks. Key issues include inadequate multi-physics coupling, static tracking, and historical context forgetting [8,9,10].

### 1.1. Related Work and Research Gaps

Quantitative pipeline risk assessment requires precise spatial and temporal integration of heterogeneous data. Historically, the industry relied on manual data alignment using physical anchors. This approach is inherently labor-intensive and unscalable. Rule-based methods (e.g., linear rubber-sheeting) accelerate this process. However, they fail catastrophically in environments with high noise or non-linear wheel slippage.

Researchers increasingly adopt deep learning to enable quantitative risk assessment [11,12]. However, pipeline degradation is fundamentally a long-term time-series forecasting problem. Recurrent networks (RNNs, LSTMs) address sequential data but suffer from limited context windows. This makes them prone to historical context forgetting. Large foundation models offer episodic memory capabilities [13,14]. Yet, managing operational data in a centralized cloud violates strict industrial privacy regulations. Federated Learning (FL) mitigates privacy concerns by keeping data localized [15,16,17]. Still, standard FL struggles with severe statistical heterogeneity across diverse geological conditions.

Multi-Agent Systems (MAS) offer a superior decentralized architecture for industrial intelligence [18,19]. Recent conceptual advancements leverage Large Language Models (LLMs) and Retrieval-Augmented Generation (RAG). Despite this, the practical deployment of MAS in pipeline integrity management remains in its infancy [20].

Explicit Research Gaps: Existing methodologies fail to seamlessly integrate decentralized multi-source data while enforcing physical engineering constraints. Furthermore, they struggle to mitigate historical forgetting and ensure decision interpretability under strict data privacy regulations. Table 2 systematically compares these existing frameworks.

### 1.2. Proposed Framework and Contributions

**Figure 2:**
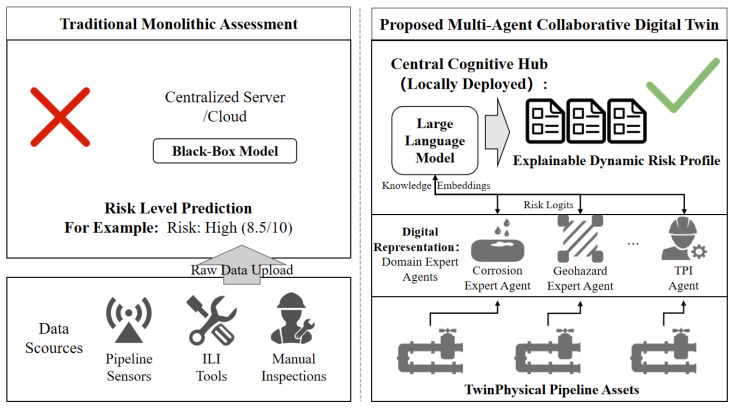
Architectural Evolution to a Multi-Agent Collaborative Digital Twin. This diagram explicitly delineates physical assets from their digital representations. It contrasts traditional models with the proposed decentralized architecture, where edge expert agents collaborate with a locally deployed, privacy-preserving cognitive hub for explainable risk analysis. Key advantages and computational limitations are also highlighted.

This paper proposes an evolutionary digital twin framework driven by a Small-Large Model Collaborative (SLMC) architecture. This framework explicitly bridges the aforementioned research gaps. As illustrated in Figure 2, it represents an architectural evolution from centralized black-box AI to a decentralized collaborative ecosystem.

The proposed framework uniquely achieves a comprehensive balance of precision, transparency, and privacy. It strictly adheres to physical engineering boundaries at the edge. It ensures highly transparent decision-making via cognitive reasoning. Finally, it achieves continuous evolutionary learning without compromising industrial data privacy. The main contributions are summarized as follows:

- Novel SLMC Digital Twin Architecture: We propose a decentralized multi-agent framework. It bridges domain-specific edge physical models with the cognitive reasoning of a central hub.
- Robust Spatio-Temporal Alignment: We design an intelligent alignment agent using dynamic time warping and weighted least squares. This overcomes data silos to establish a mathematically unified spatial ground truth.
- Synergistic Continuous Evolutionary Learning: The framework establishes a cross-scale feedback loop linking cognitive reasoning, human-in-the-loop validation, and EWC parameter protection. This dynamically updates the knowledge base and mitigates catastrophic forgetting.

The remainder of this paper is organized as follows. Section 2 defines the data alignment problem and SLMC mechanisms. Section 3 presents experimental results. Section 4 discusses engineering implications and limitations. Section 5 concludes the paper.

## 2. Materials and Methods

Distributed and dynamic digital architectures are essential for managing complex pipeline networks [21,22]. This section introduces proprietary datasets and defines core engineering challenges via mathematical formulations. It also details the structural mechanisms of the proposed Small-Large Model Collaborative (SLMC) architecture [23,24].

### 2.1. Datasets and Problem Formulation

This study constructs a proprietary dataset from two operational pipelines (SJ and LC) for rigorous framework evaluation. The SJ Pipeline traverses complex mountainous and river-crossing terrains. Having operated for over 15 years, it faces severe corrosion and geohazard threats. Conversely, the LC Pipeline is a newer urban-fringe network. It experiences dynamic changes in high-consequence areas and frequent third-party interference. Table 3 summarizes the quantitative statistics of these datasets.

The primary datasets contain sensitive national energy infrastructure information. They are subject to strict non-disclosure agreements with PipeChina. Consequently, raw operational logs and exact coordinates remain confidential due to safety restrictions. However, an anonymized sample dataset and the core multi-agent code are available upon reasonable request. This ensures scientific reproducibility without compromising infrastructure security.

The pipeline network is mathematically abstracted as a directed spatial graph G=(V,E). The node set V represents critical infrastructure points (e.g., pump stations, valve chambers). The edge set E represents physical pipeline connections. A highly dimensional feature space influences the pipeline state over time. The ensuing operational challenges are defined via three interrelated problem formulations.

First, spatial–temporal data alignment resolves heterogeneities in multi-modal data D. These disjointed streams include in-line inspections, pressure telemetry, drone imagery, and geotechnical data. Each source utilizes different spatial reference frames and sampling frequencies. The objective is to find an optimal mapping function $f_{align}$. This function projects raw coordinates onto a standardized anchor space $S_{anchor}$ by minimizing spatial projection errors:

(1) $$\min_{f_{align}}\sum_{m\in D}\left|\right|f_{align}\left(x_{m},t_{m}\right)-S_{anchor}^{*}{\left|\right|}^{2},$$

where $S_{anchor}^{*}$ is the ground-truth physical location. Variables $x_{m}$ and $t_{m}$ represent raw spatial and temporal coordinates from modality *m*.

Second, dynamic risk assessment formulates pipeline degradation as a continuous process rather than a static classification task. It requires a predictive function $F_{risk}$ mapping aligned multivariate sequences $X_{t}^{\left(i\right)}$ to a future risk profile $R_{t+\Delta t}^{\left(i\right)}$. This profile vector represents probabilities and severities across multiple failure modes:

(2) $$R_{t+\Delta t}^{\left(i\right)}=F_{risk}\left(X_{t}^{\left(i\right)},X_{t-1}^{\left(i\right)},\ldots ,X_{t-k}^{\left(i\right)};\Theta_{expert}\right),$$

where $\Theta_{expert}$ denotes parameters of domain-specific physical models (e.g., corrosion kinetics or geohazard stress).

Third, intelligent decision-making is formulated as a Constrained Markov Decision Process. The digital twin must translate quantified risks into actionable engineering interventions. The objective is to find an optimal policy $\pi^{*}$. This policy maps the global state space to discrete intervention actions, maximizing safety utility *U* over a horizon *T*:

(3) $$\pi^{*}=\arg\max_{\pi }E\left[\sum_{t=0}^{T}\gamma^{t}U\left(S_{t},A_{t}\right)\right],$$

subject to the resource constraint $\sum C\left(A_{t}\right)\leq C_{max}$ (maximum maintenance budget) and the critical safety boundary $R\left(S_{t}|A_{t}\right)\leq R_{threshold}$ (strict industry regulatory standards).

### 2.2. The Proposed SLMC Architecture

**Figure 3:**
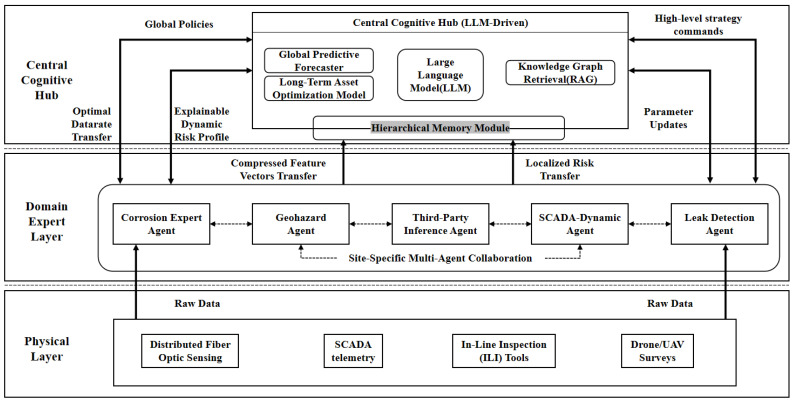
Topological Architecture of the Multi-Agent Digital Twin System. The framework integrates (1) the Physical Layer for multi-modal sensor data acquisition, (2) the Domain Expert Layer for decentralized physics-informed risk assessment, and (3) the Central Cognitive Hub for LLM-driven decision synthesis.

This research transitions pipeline assessment from monolithic black-box models to a distributed cognitive network. Traditional centralized systems suffer from bandwidth limits, high latency, and strict privacy regulations. As illustrated in Figure 3, the proposed architecture balances localized edge computing with global cognitive reasoning. It utilizes a hierarchical multi-agent ecosystem. Physics-informed industry models are encapsulated as Domain Expert Agents. Simultaneously, a locally deployed Large Language Model acts as the central hub. This synergy enables the autonomous execution of complex reasoning and observation tasks.

The structural design ensures that high-bandwidth raw data is processed locally at the edge, while only compressed, semantic risk information is transmitted to the central cognitive hub [20]. This hierarchical topology not only preserves data privacy by keeping raw operational logs at the local nodes but also significantly reduces the communication latency typical of centralized cloud architectures [15]. Within this framework, the Domain Expert Agents serve as the high-fidelity perception engine, while the LLM-driven hub acts as the system’s brain, resolving conflicting expert reports and ensuring that all maintenance decisions strictly comply with engineering standards and safety regulations [13].

### 2.3. Intelligent Data Standardization and Spatial Alignment Agent

**Figure 4:**
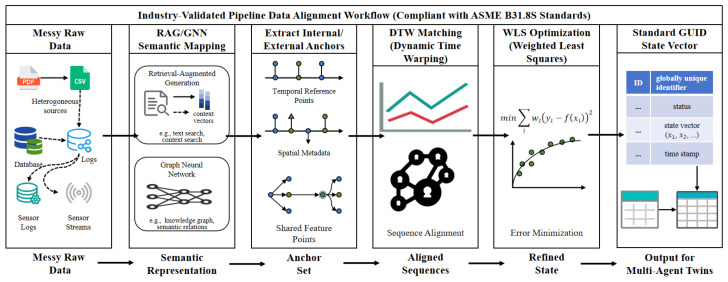
ASME B31.8S-Compliant Data Alignment Workflow. This process establishes a unified spatial ground truth for heterogeneous logs, resolving data silos via dynamic time warping and weighted least squares.

To circumvent the limitations of confronting synonym ambiguity and semi-structured texts, the agent constructs an intelligent transformation workflow based on a configurable Retrieval-Augmented Generation knowledge base (illustrated in Figure 4).

The alignment agent resolves heterogeneity by modeling anchor mileage $s_{i}$ as a Gaussian variable $s_{i}∼N\left(\mu_{i},\sigma_{i}^{2}\right)$. Operational data exhibits severe formatting, semantic, and spatial disparities. During feature engineering, a Sequence-to-Sequence model with Conditional Random Fields extracts structured entities from complex fields. For precise mapping, a semantic Graph Neural Network calculates confidence scores between raw and standard fields. Finally, hierarchical clustering enables unsupervised identification of unknown patterns to dynamically expand the knowledge base.

To enhance robustness against inherent cumulative errors from odometer wheel slippage and variable GPS precision, the system introduces a dynamic weight allocation mechanism based on a Data Quality Score DQS, where the optimized variance is defined as $\sigma_{i}^{opt}=\sigma_{i}\cdot \left(1+\lambda \cdot \left(1-DQS_{i}\right)\right)$. Low-quality data points artificially amplify their variance, automatically reducing their constraint weight in subsequent alignment calculations and thereby significantly enhancing algorithmic robustness.

For internal sequence matching between disparate inspection runs, the agent establishes a candidate set via Dynamic Time Warping to overcome non-linear mileage deviations. Subsequently, it applies a Weighted Least Squares optimization to minimize the residual errors of the matched pairs, solving for linear stretch and translation coefficients:

(4) $$\min_{a,b}\sum_{\left(i,j\right)\in M_{cand}}\frac{1}{{\left(\sigma_{A,i}^{opt}\right)}^{2}+{\left(\sigma_{B,j}^{opt}\right)}^{2}}\cdot {\left(\left(a\cdot s_{A,i}+b\right)-s_{B,j}\right)}^{2}.$$

For absolute spatial correction utilizing high-precision external test piles, a piece-wise linear stretching model adaptively calibrates local cumulative errors across segments defined by adjacent test piles *k* and k+1:

(5) $$f\left(s_{A}\right)=s_{B,k}+\frac{s_{B,k+1}-s_{B,k}}{s_{A,k+1}-s_{A,k}}\cdot \left(s_{A}-s_{A,k}\right).$$

This rigorous mathematical optimization outputs a standardized state vector mapped to a global unique identifier.

### 2.4. Edge Execution Layer: Domain Expert Agents

**Figure 5:**
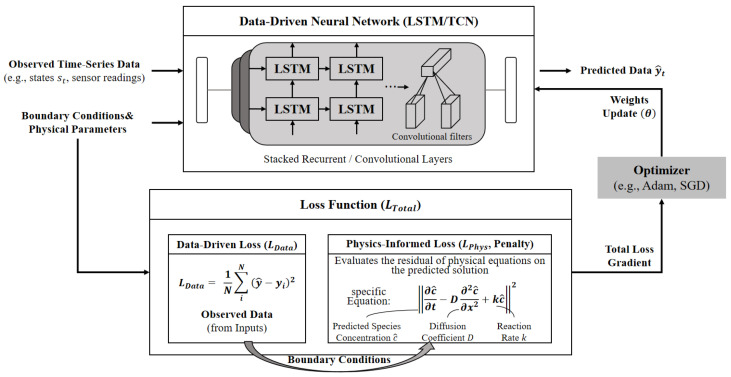
The Physics-Informed Neural Network (PINN) Core of Expert Agents. Data-driven neural networks constrained by physics-informed loss functions to ensure thermodynamic compliance.

Domain Expert Agents act as decentralized execution units constructed upon Physics-Informed Neural Network architectures (depicted in Figure 5).

These agents maintain strict adherence to physical laws by embedding governing equations directly as penalty terms during optimization. This dual-driven data and physics mechanism ensures that the generated local risk logits strictly converge within thermodynamic boundaries, drastically enhancing the model’s cross-cycle generalization capability.

#### 2.4.1. Corrosion and Structural Integrity Agent

This agent utilizes a Physics-Informed LSTM to forecast metal loss and remaining useful life. To mitigate the measurement noise inherent in harsh pipeline environments, the agent first applies an Unscented Kalman Filter to dynamically smooth the magnetic flux leakage signals. Formally, the discrete-time non-linear degradation state transition is modeled as $x_{k}=f\left(x_{k-1}\right)+w_{k-1}$ with sensor observation $z_{k}=h\left(x_{k}\right)+v_{k}$, where $w_{k-1}$ and $v_{k}$ represent the process and measurement covariance noise matrices, respectively. The theoretical physical degradation rate CR(t), driven by soil resistivity ρ, pH, temperature *T*, and fluid velocity *v* based on electrochemical corrosion kinetics including Faraday laws, is mathematically formalized as $CR\left(t\right)=\frac{M\cdot i_{corr}\left(t,\rho ,pH,T,v\right)}{n\cdot F\cdot \rho_{m}}$, where $i_{corr}$ denotes the dynamic corrosion current density, *M* is the molar mass of the pipeline steel, *n* is the number of transferred electrons, *F* is Faraday’s constant, and $\rho_{m}$ is the metal density. This physical constraint is incorporated into the loss function as a soft penalty regularization:

(6) $$L_{expert}=\frac{1}{N}\sum_{i=1}^{N}{∥{\hat{d}}_{i}-d_{i}∥}^{2}+\lambda {∥\frac{\partial \hat{d}}{\partial t}-CR\left(t,\rho ,pH,T,v\right)∥}^{2},$$

where ${\hat{d}}_{i}$ is the predicted defect depth and $d_{i}$ is the actual benchmark obtained via excavation. This dual-driven mechanism ensures that risk logits converge within thermodynamic boundaries.

#### 2.4.2. Geohazard and Environmental Threat Agent

Operating as the dynamic spatial risk evaluator, this agent focuses on external environmental threats including landslides, surface subsidence, and seismic fault activities. The agent deeply fuses millimeter-level surface deformation time series provided by persistent scatterer radar interferometry with high-precision digital elevation models generated via drone photogrammetry. The agent calculates a dynamic Geohazard Vulnerability Index GVI by integrating multi-modal data through a multi-factor non-linear aggregation:

(7) $$GVI\left(x,y,t\right)=\int_{t_{0}}^{t}\sum_{k=1}^{K}\omega_{k}\left(\tau \right)\cdot \psi_{k}\left(x,y,\tau \right)\cdot \Phi \left(\sigma_{pipe},\epsilon_{soil}\right)d\tau ,$$

where $\Phi (\sigma_{pipe},\epsilon_{soil})$ introduces a Finite Element Surrogate model based on the Mohr-Coulomb failure criterion, rigorously defined by the yield surface $f_{MC}=\tau -\sigma_{n}\tan\phi -c\leq 0$, where τ is the induced shear stress, $\sigma_{n}$ is the normal stress, ϕ is the angle of internal friction, and *c* is the soil cohesion. This surrogate function Φ acts as an extremely fast non-linear mapping for soil displacement $\epsilon_{soil}$ to additional pipe bending stress $\sigma_{pipe}$. This enables the transition from static geographical descriptions to dynamic structural stress early warnings.

#### 2.4.3. Leakage and High Consequence Area Agent

This agent evaluates dynamic spatial consequences by integrating a CFD-based Reduced Order Model [8]. Addressing the leakage and dispersion of high-pressure mediums, the agent abandons the static coarse circular potential impact radius. Instead it incorporates a real-time Gaussian Plume dispersion algorithm accessing telemetry including wind speed, atmospheric stability, and dynamic operating pressure to generate an asymmetric drifting thermal radiation or toxic dispersion boundary. Specifically, at ground level (z=0), the instantaneous spatial concentration distribution is formalized as $D\left(x,y,t\right)=\frac{Q(t)}{\pi u\sigma_{y}\sigma_{z}}\exp\left(-\frac{y^{2}}{2\sigma_{y}^{2}}-\frac{H_{eff}^{2}}{2\sigma_{z}^{2}}\right)$, where Q(t) represents the dynamic leak rate driven by internal pressure transients, *u* is the mean downwind velocity, $H_{eff}$ is the effective release height, and $\sigma_{y},\sigma_{z}$ are the Pasquill-Gifford atmospheric dispersion parameters. The comprehensive risk value $HCA_{risk}$ is calculated via spatial integration:

(8) $$HCA_{risk}={\;\int \int \;}_{\Omega_{disp}}D\left(x,y,t\right)\cdot P_{dyn}\left(x,y,t\right)\cdot V_{infra}\left(x,y\right)dxdy,$$

where D(x,y,t) is the dynamic radiation intensity and $P_{dyn}$ is the time-varying population density field derived from cellular mobility data. This mechanism guarantees that the system accurately identifies tidal commuting corridors characterized by low risk at night and high risk during the day.

### 2.5. Central Cognitive Agent and Continuous Learning

The Central Cognitive Agent translates expert heuristic knowledge into a computable State-Action-Reason triplet model $E_{exp}=\langle S_{c},A_{opt},R_{logic}\rangle$ [24]. Formally, at decision step *t*, the global state space $S_{c}$ is constructed by concatenating the high-dimensional risk embeddings output by the *N* decentralized edge expert agents: $S_{t}={⨁}_{i=1}^{N}\phi_{i}\left(X_{t}^{\left(i\right)}\right)$, where $\phi_{i}$ denotes the embedding function of the *i*-th agent. To enable cognitive processing, this continuous state vector is projected into the discrete vocabulary space of the LLM and concatenated with a standardized system instruction prompt I and the retrieved context $C_{t}$ to form the unified input sequence $P_{t}=\left[I⊕S_{t}⊕C_{t}\right]$.

To ensure computational reproducibility and strictly mitigate data privacy risks, the cognitive hub is powered by a locally deployed Llama-3-70B-Instruct (Meta Platforms, Inc., Menlo Park, CA, USA) model operating entirely within an air-gapped environment. This guarantees that the system absolutely does not rely on external cloud services or internet connectivity, ensuring zero risk of sensitive operational data leakage. The model is quantized to 4-bit precision to optimize the trade-off between reasoning depth and inference latency. All cognitive fusion experiments were conducted on a localized workstation equipped with dual NVIDIA H100 (80 GB) GPUs (NVIDIA Corporation, Santa Clara, CA, USA) and 256 GB of RAM. Furthermore, the system instruction prompt I is engineered to strictly confine the model to an “Act as a Senior Pipeline Integrity Engineer” persona, enforcing a deterministic Reasoning-Action output structure.

Utilizing natural language processing dependency parsing techniques, the system explicitly extracts fuzzy operational justifications from expert logs into a Causal Logic Graph transforming human intuitive experience into dense embeddings. As detailed in Table 4, this structure allows the system to synthesize trade-offs between economic costs and safety redundancy [19].

**Figure 6:**
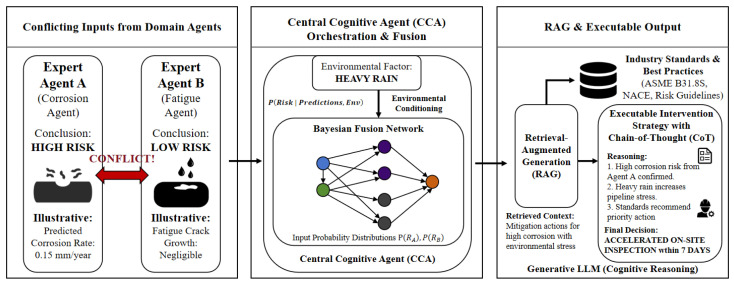
Central Cognitive Agent Orchestration and Conflict Resolution. Conflicting edge inputs are resolved via Bayesian fusion and Retrieval-Augmented Generation.

For novel risks, the Central Agent computes the Graph Edit or Wasserstein distance between the current state and retrieved historical references. To ensure interpretability and minimize LLM hallucinations, the reasoning engine integrates an Industry Knowledge Graph Retrieval-Augmented Generation (RAG) framework. This hybrid architecture pairs dense semantic matching with sparse regulatory code retrieval. Decision generation follows a Directed Acyclic Graph (DAG) Chain-of-Thought process: phenomenon analysis, historical matching, attribution reasoning, and final output (Figure 6).

Mathematically, the LLM ($\theta_{LLM}$) models the conditional probability of generating the Action-Reason sequence $Y_{t}=\langle A_{opt},R_{logic}\rangle$ autoregressively:

(9) $$P\left(Y_{t}|P_{t};\theta_{LLM}\right)=\prod_{l=1}^{|Y_{t}|}P_{\theta_{LLM}}\left(y_{l}|y_{<l},P_{t}\right).$$

Decision optimization relies on a multi-objective reward $r\left(S_{t},A_{t}\right)=\omega_{1}U_{safety}-\omega_{2}C_{cost}+\omega_{3}\delta_{expert}$, balancing the safety margin ($U_{safety}$), economic cost ($C_{cost}$), and discrete Human-in-the-Loop validation ($\delta_{expert}\in \left\{-1,1\right\}$). The agent policy is optimized via Proximal Policy Optimization (PPO), driven by the task loss $L_{curr}\left(\theta_{LLM}\right)$:

(10) $$L_{curr}\left(\theta_{LLM}\right)=-E_{P_{t}}\left[\min\left(\rho_{t}\left(\theta_{LLM}\right){\hat{A}}_{t},\mathrm{clip}\left(\rho_{t}\left(\theta_{LLM}\right),1-\epsilon ,1+\epsilon \right){\hat{A}}_{t}\right)\right],$$

where $\rho_{t}\left(\theta_{LLM}\right)=\frac{\pi_{\theta_{LLM}}\left(A_{t}|P_{t}\right)}{\pi_{\theta_{old}}\left(A_{t}|P_{t}\right)}$ denotes the probability ratio and ${\hat{A}}_{t}$ is the generalized advantage estimator.

**Figure 7:**
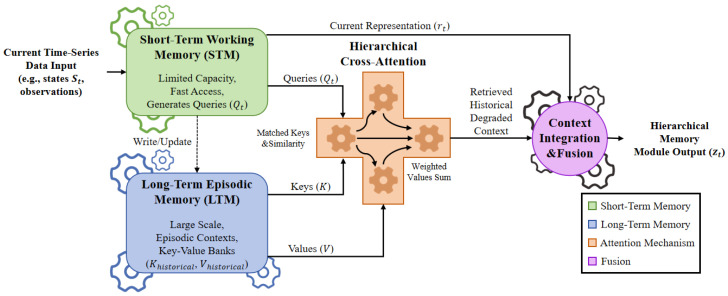
Structure of the Hierarchical Time-Series Memory Module. Short-Term Working and Long-Term Episodic memories use cross-attention to retrieve historical degradation contexts.

Continuous feedback learning utilizes a hierarchical memory (Working, Episodic, and Semantic). The module retrieves historical context for ongoing risk evaluation via cross-attention:

(11) $$Context_{t}=\mathrm{Attention}\left(q_{t},K_{hist},V_{hist}\right),$$

where $q_{t}$ represents the current query and $K_{hist}$ denotes historical episodic keys (Figure 7).

The Semantic Memory physically manifests as an Expert Decision Graph which executes online incremental learning via three topological evolution mechanisms including cognitive node growth, knowledge consolidation fusion, and spatio-temporal adaptive pruning.

**Figure 8:**
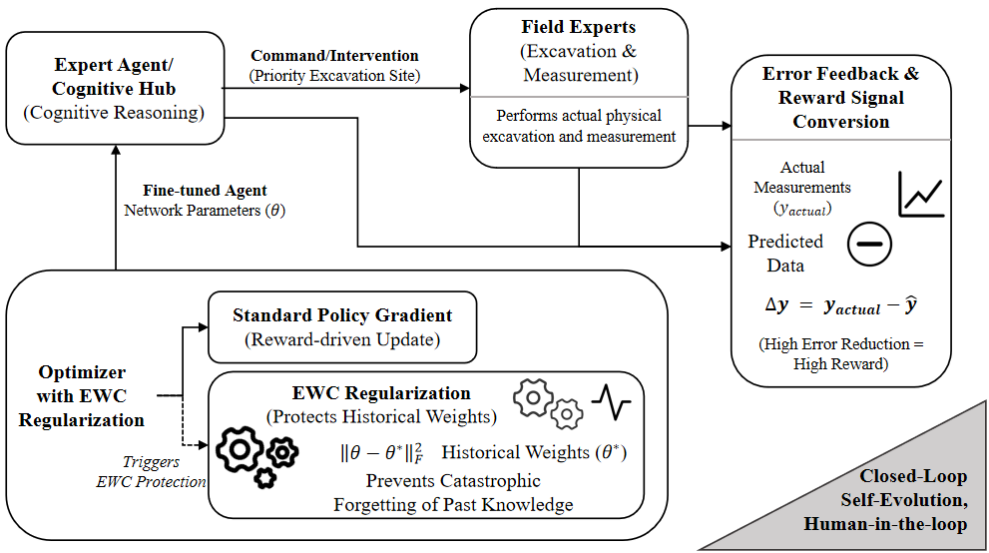
Continuous Feedback Learning Loop with Elastic Weight Consolidation (EWC). Human-in-the-loop feedback drives policy fine-tuning while EWC prevents catastrophic forgetting.

To prevent catastrophic forgetting during continuous adaptation to evolving physical conditions, the framework utilizes Elastic Weight Consolidation, augmenting the loss function with a Fisher Information-based regularization term [10]:

(12) $$L\left(\theta \right)=L_{curr}\left(\theta \right)+\frac{\lambda }{2}\sum_{i}F_{i}{\left(\theta_{i}-\theta_{i,old}\right)}^{2},$$

where $F_{i}$ ensures parameter stability by penalizing shifts in weights critical to historical pipeline degradation patterns. The system operates as a human-in-the-loop closed cycle where the cognitive hub issues intervention commands to field experts whose actual physical measurements generate an error-based reward signal driving policy gradient fine-tuning (illustrated in Figure 8).

Crucially, the primary scientific innovation of this architecture extends beyond the mere integration of existing AI components. The proposed framework establishes a synergistic Continuous Feedback Learning mechanism explicitly tailored to pipeline integrity management. By mathematically coupling the cognitive reasoning of the central LLM with the physical validation of human experts through the PPO reward signal, and dynamically utilizing this feedback to trigger EWC parameter protection, the system creates a rigorous cross-scale feedback loop. This coupled innovation ensures that localized physical constraints directly guide the global cognitive evolution, effectively resolving the fundamental dilemma between task plasticity and historical stability inherent in long-term infrastructure management.

### 2.6. Experimental Baselines and Setup

To benchmark performance, Table 5 outlines the selected baseline methods targeted across different execution phases to rigorously validate the superiority of the proposed architecture against established algorithms and recent highly competitive models.

For spatial alignment tasks, manual alignment represents the traditional industry standard which is highly accurate but computationally inefficient. Linear rubber-sheeting distributes cumulative odometer errors linearly but fails to address non-linear wheel slippage. For corrosion prediction, empirical models provide strong interpretability but lack capacity to map non-linear environmental degradation factors, whereas standard data-driven deep networks demonstrate significant variance violating thermodynamic boundaries. For global decision synthesis, monolithic multi-layer perceptrons struggle with heterogeneous inputs, and standard multi-agent systems lacking a central language model fail to resolve complex multi-physics conflicts effectively.

To ensure rigorous reproducibility as demanded by industrial applications, the proprietary datasets were strictly partitioned into training (80 percent), validation (10 percent), and testing (10 percent) sets. This partitioning was executed chronologically to strictly prevent temporal data leakage. Model optimization was conducted using the AdamW optimizer. Table 6 details the core hyperparameter configurations utilized during the training phase across all deep learning baselines and the proposed framework.

## 3. Results

This section quantitatively evaluates the Small-Large Model Collaborative (SLMC) framework across four engineering dimensions. Using proprietary SJ and LC pipeline datasets, targeted experiments independently assess the perception, edge execution, cognitive, and evolutionary layers under diverse operational conditions.

### 3.1. Evaluation Metrics

The evaluation metrics align strictly with industrial safety standards. To ensure real-world applicability, a committee of five senior engineers—each with over ten years of field experience—manually annotated and cross-validated the ground-truth labels for all risk tasks, establishing a highly rigorous, expert-labeled benchmark.

Alignment Accuracy measures the proportion of inspection features mapped within a 0.5 m tolerance of physical anchors, reflecting the standard permissible excavation error window. Exceeding this margin causes wrong-location excavations and accidental pipe strikes. Computational efficiency is quantified as the hours required to process 100 km of multi-modal data.

Root Mean Square Error (RMSE) and Mean Absolute Error (MAE) evaluate continuous corrosion depth predictions. RMSE is prioritized; its quadratic penalty prevents catastrophic underestimates of severe metal loss, maintaining model sensitivity to extreme degradation.

Precision, Recall, and the F1-Score evaluate the cognitive hub’s ability to classify compounded risks. Precision maximizes economic efficiency (minimizing false alarms), while Recall ensures catastrophic threats are detected. The F1-Score balances both objectives. The decision evaluation utilizes 500 representative multi-hazard scenarios extracted from historical logs, ensuring statistical significance for reliability testing.

RMSE Degradation quantifies catastrophic forgetting during continuous learning. A value near zero indicates the model successfully integrated new environmental patterns without overwriting its established historical degradation memory.

### 3.2. Spatial Alignment Performance and Efficiency

Spatial alignment is evaluated on the noisy, mountainous SJ pipeline and the newer, urban-fringe LC pipeline datasets.

Manual alignment provides a reliable benchmark (>96% accuracy) but is highly inefficient, requiring 48.0 h per 100 km for manual cross-referencing. This intense labor renders it entirely unscalable for extensive national networks.

Linear rubber-sheeting algorithms distribute errors evenly, failing significantly on the complex SJ dataset (62.4% accuracy) due to severe non-linear wheel slippage in steep terrains. While performing slightly better on the LC dataset (75.3%), this method remains insufficient for precise integrity management.

Vanilla dynamic time warping (DTW) accommodates non-linear distortions but struggles with high signal noise, achieving 81.2% (SJ dataset). Lacking probabilistic weighting, it fails in harsh conditions.

**Figure 9:**
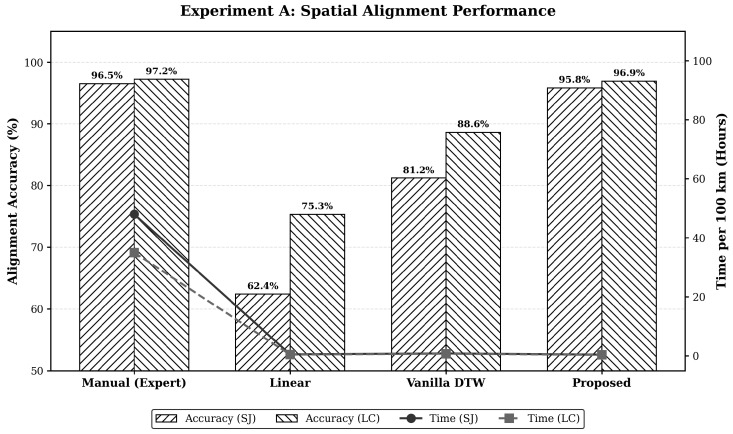
Performance comparison of spatial alignment methods across heterogeneous datasets. The dual-axis visualization demonstrates that the proposed method approaches expert-level accuracy while suppressing computational time to sub-hour levels.

The proposed agent explicitly resolves the accuracy–efficiency trade-off. Combining DTW with weighted least squares, it achieves highly competitive operational precision (95.8% on SJ; 96.9% on LC) in merely 0.3–0.4 h (Figure 9). This sub-hour processing establishes a unified spatial ground truth for the downstream digital twin.

### 3.3. Domain Expert Agent Predictive Accuracy

To validate the necessity of embedding physical laws into edge-computing nodes, the corrosion expert agent was evaluated over a five-year operational horizon. This experiment assesses the predictive precision of the domain expert agent against traditional empirical and purely data-driven models. The evaluation spans both the severely degraded SJ pipeline and the relatively newer LC pipeline datasets to ensure comprehensive validation.

As shown in Table 7, traditional empirical models based on modified industry standards exhibit the highest errors across both datasets. They record a root mean square error of 3.45 on the SJ pipeline and 2.95 on the LC pipeline. These models rely on static mathematical formulas that completely ignore dynamic environmental coupling effects such as fluctuating soil moisture and variable pH levels over the pipeline lifecycle.

Conversely, purely data-driven models like random forest and standard long short-term memory networks show significant variance and high error margins. To comprehensively evaluate highly competitive time-series architectures, recent Transformer-based foundation models, including One Fits All (GPT4TS) and Chronos, were integrated into the comparative analysis. As detailed in Table 7, these advanced attention-based models outperform traditional LSTMs.

**Figure 10:**
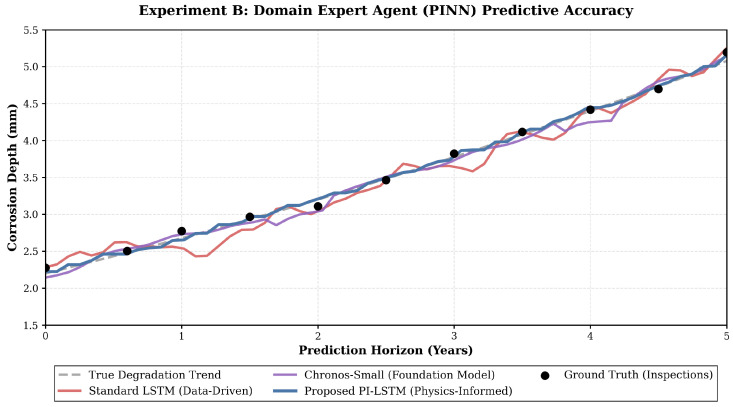
Trajectory of localized corrosion depth prediction over a five-year horizon. The proposed physics-informed agent strictly enforces a monotonically increasing degradation curve that smoothly tracks the ground truth inspections.

Notably, the Chronos-Small model demonstrates superior feature extraction capabilities with lower prediction errors compared to the Chronos-Tiny variant, accurately reflecting the expected performance gains associated with increased parameter scale. However, while they excel in multi-dimensional mapping, both traditional and foundation data-driven models fundamentally lack strict physical constraints. As vividly demonstrated in Figure 10, unconstrained neural networks frequently predict erratic trajectories that blatantly violate thermodynamic boundaries. These data-driven baselines often display physically impossible reductions in corrosion depth over time, suggesting that the metal wall is magically regenerating. Such unphysical fluctuations destroy trust in the predictive model among field engineers.

By directly embedding Faraday laws and degradation kinetics as a physics-informed penalty term, the proposed agent effectively suppresses these unphysical fluctuations. It strictly enforces a monotonically increasing degradation curve that smoothly tracks the true degradation trend and aligns with ground truth inspections. This dual-driven mechanism ensures that the generated risk logits strictly converge within thermodynamic boundaries. Consequently, the proposed agent consistently achieves the lowest errors, reducing the root mean square error to 2.14 on the complex SJ dataset and 1.86 on the LC dataset. This dual-dataset validation proves that the collaborative domain expert agent serves as a highly robust and physically consistent computational engine for both early-stage and late-stage pipeline degradation prediction.

### 3.4. Global Cognitive Decision Synthesis and Conflict Resolution

The central cognitive agent capability to synthesize conflicting edge reports and formulate holistic intervention strategies is thoroughly evaluated. One of the primary concerns in deploying large language models (LLMs) for critical infrastructure is the phenomenon of hallucination, where the model may generate plausible but physically incorrect or non-compliant decisions. In this framework, hallucinations are systematically suppressed through two primary mechanisms. First, the reasoning engine is strictly constrained by an Industry Knowledge Graph-based RAG module, which forces the model to ground its decisions in actual regulatory codes (e.g., ASME B31.8S). Second, the outputs from the edge expert agents act as physics-informed “hard boundaries”; any decision that violates the thermodynamic convergence of the PINN models is robustly filtered during the Bayesian fusion phase. To rigorously test the reasoning generalization of the large language model hub, the evaluation is conducted across the SJ pipeline dominated by natural multi-physics threats such as severe corrosion coupled with imminent landslide stress, and the LC pipeline dominated by socio-environmental threats such as simultaneous third-party vibrations in densely populated urban fringes.

As detailed in Table 8, the monolithic multi-layer perceptron struggles significantly to process multi-modal heterogeneous inputs across both domains. It yields suboptimal F1-scores of 73.1 percent and 76.5 percent respectively, representing the limitations of a traditional centralized black-box paradigm. The recent knowledge graph neural network baseline performs aggressively and achieves the highest recall, reaching up to 94.5 percent on the LC pipeline. However, this high recall comes at a severe cost of precision, dropping to 82.5 percent on the SJ dataset. Lacking dynamic causal reasoning, the knowledge graph model overreacts to correlated but harmless entities, generating numerous costly false alarms that would severely drain maintenance resources.

In contrast, the central hub employs Bayesian fusion and Retrieval-Augmented Generation (RAG) to filter localized noise. This yields highly competitive precision and peak F1-scores across both risk domains. Specifically, achieving a 94.2% F1-Score and 95.1% precision on the LC pipeline demonstrates expert-level reasoning that effectively minimizes costly false excavations. By resolving expert conflicts, the cognitive hub maintains rigorous safety thresholds without unnecessary operational disruptions.

To further validate the architectural necessity of the individual components within the SLMC framework, an ablation study was conducted on the decision synthesis task. As presented in Table 9, the study quantifies the impact of removing the Retrieval-Augmented Generation (RAG) module and the Physics-Informed Neural Network (PINN) constraints. The evaluation introduces an additional metric, Physical Consistency, which measures the percentage of generated decisions that strictly adhere to thermodynamic and physical boundaries.

The ablation results clearly indicate that both components are indispensable for reliable infrastructure management. Removing the RAG module primarily degrades reasoning precision (dropping from 95.1 percent to 86.4 percent), as the LLM loses grounding in strict regulatory codes and is prone to hallucinating overly conservative or non-compliant interventions. Conversely, removing the PINN edge constraints leads to a catastrophic drop in Physical Consistency (from 99.8 percent to 74.2 percent). Without these edge-computed physical hard boundaries, the central hub frequently accepts unphysical predictions from pure data-driven models, highlighting the critical role of physics-informed constraints in filtering invalid decisions.

**Figure 11:**
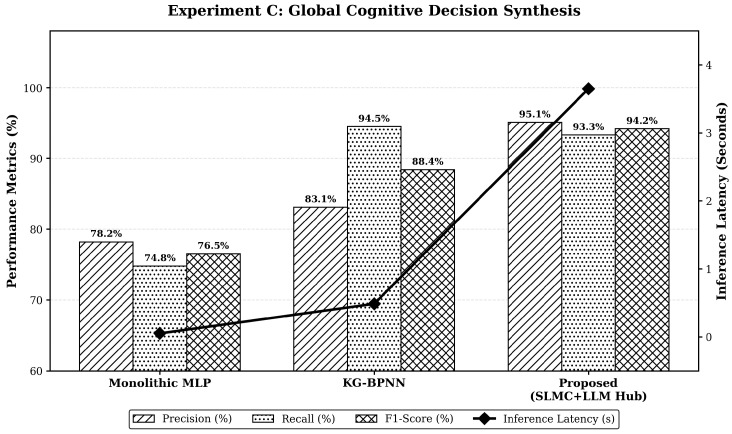
Multi-objective trade-off analysis of global cognitive decision synthesis. The dual-axis chart visualizes the engineering compromise between Precision, F1-Score, and inference latency.

It is critical to acknowledge the inherent computational and operational trade-offs of this architectural choice, which are starkly visualized in Figure 11.

First, the proposed method yields a marginally lower recall compared to the highly aggressive knowledge graph baseline. This occurs because the strict chain-of-thought reasoning process actively dismisses borderline anomalies to prevent costly false excavations. Secondly, the inclusion of semantic reasoning introduces a significant computational overhead. The inference latency of the hub reaches 3.50 s per complex event fusion, which is vastly inferior to the 0.05 s latency of a standard multi-layer perceptron.

Nevertheless, within the context of pipeline integrity management where maintenance dispatching is typically planned over hours or days rather than milliseconds, this latency is an entirely acceptable compromise. The deliberate acceptance of a substantially higher inference latency guarantees rigorous and standard-compliant semantic reasoning, representing a massive gain in decision transparency and operational reliability.

### 3.5. Continuous Learning and Anti-Forgetting Evaluation

To evaluate the evolutionary aspect of the digital twin, the final experiment investigates the system capacity for continuous feedback learning across sequential operational domains. The evaluation protocol utilizes both proprietary datasets chronologically. The model is initially trained on the SJ pipeline focusing on long-term mountainous corrosion features. It is subsequently fine-tuned on the LC pipeline focusing on socio-environmental interference and urban dynamic risks.

Experiment results for catastrophic forgetting mitigation are systematically shown in Table 10. These results perfectly illustrate the classic stability versus plasticity dilemma in continuous machine learning. When utilizing the standard fine-tuning baseline, the model exhibits high plasticity, learning the new LC pipeline features exceptionally well and achieving a minimal root mean square error of 1.95. However, this unconstrained parameter updating severely degrades the neural weights associated with historical knowledge. When the standard fine-tuning model is re-evaluated on the original SJ pipeline dataset, the error spikes drastically from 2.14 to 4.35. This massive performance drop confirms the occurrence of severe catastrophic forgetting, rendering the standard fine-tuning approach unacceptable for long-term infrastructure management.

By implementing elastic weight consolidation within the continuous feedback loop, the proposed framework explicitly addresses this vulnerability. The system calculates the Fisher information matrix to quantify and strictly protect the critical parameters corresponding to the original SJ dataset. Consequently, after training on the new pipeline segment, the proposed method successfully retains its historical memory, maintaining an exceptional root mean square error of 2.28 on the original dataset.

In keeping with rigorous architectural evaluation, a necessary engineering trade-off is observed. Because the elastic weight consolidation regularization restricts the parameter search space to preserve historical weights, the model predictive accuracy on the newly introduced LC pipeline is marginally inferior to that of the unconstrained standard fine-tuning baseline. However, in the context of lifecycle pipeline integrity, sacrificing a fractional margin of accuracy on a new segment to reliably preserve decades of historical degradation logic across the entire pipeline network is an indispensable and highly advantageous engineering compromise. This mechanism ensures the digital twin can safely evolve over decades of continuous operation.

## 4. Discussion

The results obtained from the multi-agent SLMC framework provide significant insights into the transition from monolithic diagnostics to collaborative intelligence. The findings confirm that integrating physical constraints with semantic reasoning resolves the most persistent bottlenecks in pipeline integrity management.

### 4.1. Scientific Interpretation of Spatial and Predictive Accuracy

The spatial alignment results indicate that cumulative mechanical drift in internal inspection tools can be effectively corrected through probabilistic anchor weighting. By approaching expert-level precision in sub-hour durations, the perception layer enables real-time digital twin synchronization. The superiority of the PI-LSTM agent over pure data-driven models suggests that physical constraints act as a critical regularization mechanism. This is particularly vital for long-distance pipelines where failure data is sparse. The enforced monotonicity in corrosion depth prediction directly prevents dangerous underestimates of metal loss, which traditional LSTMs occasionally produce due to signal noise.

### 4.2. Engineering Trade-Off Between Latency and Decision Transparency

The cognitive decision experiment revealed an essential engineering compromise. While monolithic networks offer near-instantaneous inference, they lack the transparent reasoning required for high-stakes safety. The proposed LLM hub introduces a latency of approximately 3.5 s. However, the resulting Chain-of-Thought reasoning provides human operators with a verifiable logic path, citing specific industry standards like ASME B31.8S. This transparency transforms the digital twin from a black-box tool into a collaborative advisor, reducing the likelihood of costly false excavations and improving stakeholder trust in autonomous systems.

### 4.3. Lifecycle Stability and Knowledge Retention

The successful mitigation of catastrophic forgetting addresses a major limitation of current industrial AI. Traditional models require full retraining when a pipeline is extended or new sensors are deployed, incurring massive computational costs. The SLMC architecture demonstrates that critical historical degradation logic can be preserved while adapting to new operational environments. The observed marginal loss in accuracy on the new task is a justifiable trade-off for maintaining a reliable, long-term memory of the entire asset lifecycle. This capability is foundational for the development of self-healing and evolving industrial digital twins.

## 5. Conclusions

This paper proposes an evolutionary digital twin framework driven by a Small-Large Model Collaborative (SLMC) architecture. This approach resolves the fundamental bottlenecks of long-distance pipeline integrity management. The framework explicitly decouples specialized edge numerical computation from centralized global cognitive reasoning. Consequently, it overcomes the interpretability and stability limitations of traditional monolithic artificial intelligence models.

The main contributions and experimental validations are summarized as follows:

- High-Fidelity Spatial Alignment: The intelligent perception agent achieved up to 96.9% spatial alignment accuracy. This establishes a reliable automated data foundation.
- Physics-Informed Edge Prediction: Enforcing strict adherence to thermodynamic boundaries reduced the predictive RMSE to 1.86 via embedded physics-informed neural networks.
- Transparent Cognitive Reasoning: The central cognitive hub effectively synthesized complex multi-physics conflicts. It achieved a 94.2% F1-Score while providing fully explainable decision pathways.
- Continuous Evolutionary Learning: The integration of elastic weight consolidation significantly mitigated catastrophic forgetting. This mechanism effectively balances task plasticity with historical stability.

Several limitations of the current study require acknowledgment. First, experimental validation is restricted to the specific geological and operational conditions of the SJ and LC datasets. The framework’s zero-shot generalization across environmental extremes (e.g., permafrost or offshore subsea pipelines) remains unverified. Second, relying on a massive central Large Language Model imposes significant hardware and communication latency constraints. This high-bandwidth dependency could delay emergency shutdowns in remote, network-deprived segments. Third, the cognitive hub’s decision-making evaluation relies on a classification benchmark of 500 manually annotated multi-hazard scenarios. While rigorous, this “expert-labeled” ground truth inherently carries subjective bias. It may not fully encapsulate universal industrial consensus across different corporate standards.

Future research will focus on addressing these limitations through specific directions. First, we will explore lightweight model quantization and decentralized edge-LLM deployment. This will reduce central hub latency and alleviate hardware dependencies. Second, future iterations will expand the multi-modal perception layer to ingest real-time acoustic emission sensors and drone-based visual streams. Simultaneously, we will test the framework across international pipeline networks to validate global generalizability. Additionally, future studies will incorporate consensus-based scoring frameworks, such as the Delphi method. This approach will mitigate inherent evaluation subjectivity and establish highly objective cognitive benchmarks.

Ultimately, this multi-agent cognitive ecosystem transitions pipeline risk management from reactive diagnostics into a proactive, interpretable, and self-optimizing digital infrastructure.

## Figures and Tables

**Table 1:** Analysis of Recent Major Pipeline Disasters and Traditional Technical Bottlenecks.

Incident & Year	Medium	Primary Physical Root Cause	Traditional Technical Bottlenecks & Research Gaps
Keystone Pipeline Spill Kansas, USA, 2022	Crude Oil	Bending stress fatigue compounded by historical weld anomalies and structural deformation.	Inadequate Multi-physics Coupling: Static risk models failed to dynamically synthesize pipe-soil stress interactions with historical localized defect data.
Shiyan 6.13 Explosion Hubei, China, 2021	Natural Gas	Severe localized electrochemical corrosion within a highly humid, confined urban blind-spot.	Data Silos & Static Tracking: Disconnection between physical inspection logs and dynamic urban environmental changes; failure in real-time updating.
TETCO Pipeline Rupture Kentucky, USA, 2020	Natural Gas	Degradation of historical manufacturing hard spots accelerated by long-term soil dynamics.	Historical Context Forgetting: Inability to align and project decades-old construction baseline data with modern, heterogeneous inspection signals.

**Table 2:** Comparison of Existing Pipeline Risk Assessment Frameworks.

Framework	Physical Constraint Support	Catastrophic Forgetting Mitigation	Decision Interpretability	Data Privacy Protection
Traditional Physical Models	High: Strictly governed by empirical mechanics and formulas	N/A: Static models inherently incapable of continuous learning	High: Explicit mathematical derivation processes	Low: Requires centralized gathering of raw operational data
Pure Deep Learning	Low: Purely data-driven black-box non-linear mapping	No: Highly susceptible to forgetting historical degradation patterns	Low: Lack of transparent causal reasoning mechanisms	Low: Demands massive centralized datasets for model training
Standard Federated Learning	Low: Primarily focuses on distributed statistical optimization	Partial: Alleviated via local updates but lacks structural historical memory	Low: Aggregated global neural weights remain uninterpretable	High: Raw operational data strictly remains at local edge nodes
Proposed SLMC Architecture	High: Embedded Physics-Informed Neural Networks at the edge	Yes: Achieved via Elastic Weight Consolidation and Hierarchical Memory	High: RAG-enhanced and rule-constrained reasoning	High: Transmits only semantic Risk Embeddings to the central bus

**Table 3:** Statistical Overview of the Proprietary Pipeline Datasets.

Feature/Data Type	SJ Pipeline	LC Pipeline
Total Length	150 km	100 km
Girth Welds and Physical Anchors	12,654	8320
External Geographic Anchors	142	85
Identified Corrosion Defects	7245	1520
Supervisory Control Nodes	8	6
Drone Inspection Logs	450 h	1200 h
Historical Maintenance Logs	312 cases	184 cases

**Table 4:** Formalization of the State-Action-Reason Triplet based on Engineering Logs.

Component	Data Format	Concrete Engineering Case
State	High-dimensional Vector	Physical anomaly showing localized corrosion depth at 60 percent alongside an active agricultural farming season.
Action	Hybrid Space	Reduce operational pressure by 15 percent, temporarily suspend excavation, and dispatch drone for surveillance.
Reason	Logic Embeddings	Immediate excavation incurs prohibitive farmland compensation; the pressure reduction maintains safety margins.

**Table 5:** Summary of Selected Baseline Methods for Comparative Evaluation.

Target Task	Baseline Method	Core Characteristics and Limitations	Model Type
Spatial Alignment	Linear Rubber-Sheeting	Assumes linear error distribution; fails in non-linear slippage scenarios.	Rule-based
Corrosion Prediction	Empirical Model Standard LSTM [12] Chronos (Tiny & Small) One Fits All (GPT4TS)	Physics-based but static; data-driven but prone to boundary violations. Advanced Transformer-based pre-training but fundamentally lacks physical thermodynamic constraints.	Physical Data-driven Foundation Model
Global Risk Decision	KG-BPNN [24] Standard MAS [19]	SOTA hybrid model but lacks causal reasoning; decentralized voting lacks cognitive fusion.	Hybrid Multi-Agent

**Table 6:** Experimental Setup and Core Hyperparameter Configurations. N/A: Not Applicable.

Hyperparameter	Data-Driven/PINN Models	Transformer Baselines
Optimizer	AdamW	AdamW
Learning Rate	$1\times 10^{-3}$ (Decay: 0.95)	$1\times 10^{-4}$ (Cosine Annealing)
Batch Size	64	32
Training Epochs	150 (Early Stopping: 15)	50 (Early Stopping: 10)
Loss Function Weights	$\lambda_{physics}=0.5$, $\lambda_{data}=1.0$	N/A (Pure Data-driven)
EWC Regularization	$\lambda_{EWC}=5000$	N/A

**Table 7:** Corrosion Depth Prediction Accuracy across Different Models and Datasets.

Predictive Model	Dataset	RMSE (mm)	MAE (mm)
Empirical Model modified B31G	SJ Pipeline	3.45	2.89
Random Forest (RF)	SJ Pipeline	3.12	2.65
Standard LSTM Pure Data-Driven	SJ Pipeline	2.78	2.21
Chronos-Tiny (Foundation Model)	SJ Pipeline	2.65	2.15
One Fits All (GPT4TS)	SJ Pipeline	2.50	2.02
Chronos-Small (Foundation Model)	SJ Pipeline	2.45	1.95
**Proposed Agent Core PI-LSTM**	**SJ Pipeline**	**2.14**	**1.75**
Empirical Model modified B31G	LC Pipeline	2.95	2.41
Random Forest (RF)	LC Pipeline	2.68	2.15
Standard LSTM Pure Data-Driven	LC Pipeline	2.35	1.82
Chronos-Tiny (Foundation Model)	LC Pipeline	2.20	1.75
One Fits All (GPT4TS)	LC Pipeline	2.12	1.66
Chronos-Small (Foundation Model)	LC Pipeline	2.05	1.58
**Proposed Agent Core PI-LSTM**	**LC Pipeline**	**1.86**	**1.42**

**Table 8:** Multi-Hazard Decision Synthesis Performance and Trade-offs across Heterogeneous Datasets.

Decision Architecture	Dataset	Precision (%)	Recall (%)	F1-Score (%)	Latency (s)
Monolithic MLP	SJ Pipeline	74.2	72.1	73.1	**0.05**
KG-BPNN 2024 Baseline	SJ Pipeline	82.5	**93.8**	87.8	0.52
Standard MAS Majority Voting	SJ Pipeline	84.1	83.5	83.8	0.15
**Proposed SLMC with LLM**	**SJ Pipeline**	**94.6**	92.8	**93.7**	3.80
Monolithic MLP	LC Pipeline	78.2	74.8	76.5	**0.05**
KG-BPNN 2024 Baseline	LC Pipeline	83.1	**94.5**	88.4	0.45
Standard MAS Majority Voting	LC Pipeline	86.5	85.2	85.8	0.12
**Proposed SLMC with LLM**	**LC Pipeline**	**95.1**	93.3	**94.2**	3.50

**Table 9:** Ablation Study on Decision Synthesis Components.

Configuration	Precision (%)	F1-Score (%)	Physical Consistency (%)
**Full SLMC Framework**	**95.1**	**94.2**	**99.8**
w/o RAG (Knowledge Hub)	86.4	85.2	98.5
w/o PINN (Edge Constraints)	91.2	89.5	74.2

**Table 10:** Catastrophic Forgetting Mitigation and Performance Trade-offs via EWC Regularization.

Training Stage	Evaluated Dataset	Metric	Standard Fine-Tuning	Proposed with EWC
1. Initial Training	SJ Pipeline Task A	RMSE	2.14	2.14
2. Fine-Tuning on LC	LC Pipeline Task B	RMSE	1.95	2.05
	SJ Pipeline Task A	RMSE	4.35	2.28

## Data Availability

The data presented in this study are available on request from the corresponding author. The data are not publicly available due to privacy and corporate confidentiality restrictions.

## Abbreviations

The following abbreviations are used in this manuscript:

SLMC	Small-Large Model Collaborative
MAS	Multi-Agent Systems
LLM	Large Language Models
PINN	Physics-Informed Neural Networks
SCADA	Supervisory Control and Data Acquisition
ILI	In-Line Inspection
HCA	High Consequence Area
TPI	Third-Party Interference
DTW	Dynamic Time Warping
WLS	Weighted Least Squares
EWC	Elastic Weight Consolidation
RAG	Retrieval-Augmented Generation
GNN	Graph Neural Networks
RMSE	Root Mean Square Error
MAE	Mean Absolute Error
